# Genome-Wide Polygenic Risk Score for CKD in Individuals with *APOL1* High-Risk Genotypes

**DOI:** 10.2215/CJN.0000000000000379

**Published:** 2023-11-14

**Authors:** Ha My T. Vy, Steven G. Coca, Ashwin Sawant, Ankit Sakhuja, Orlando M. Gutierrez, Richard Cooper, Ruth J.F. Loos, Carol R. Horowitz, Ron Do, Girish N. Nadkarni

**Affiliations:** 1 Icahn School of Medicine, New York City, New York; 2 University of Alabama at Birmingham, Birmingham, Alabama; 3 Loyola University School of Public Health, Chicago, Illinois

**Keywords:** apolipoprotein L1 (APOL1), CKD, ethnic minority, genetics and development, health equity, diversity, and inclusion, kidney disease, risk factors

Apolipoprotein L1 high-risk (*APOL1* HR) genotypes—defined by the G1/G2 variants at the *APOL1* locus^1^—are increasingly being tested in clinical practice and as novel targets. However, only some individuals with *APOL1* HR have CKD or kidney failure. This indicates that along with social and clinical risk determinants, genetic background contributes to the *APOL1* HR kidney disease relationship. However, few genome-wide significant genetic variants have been identified that affect the *APOL1* HR kidney disease relationship.

A polygenic risk score (PRS) aggregates the cumulative effects of millions of common genetic variants.^2^ A recent study has shown that a linear combination of PRS and *APOL1* genotype enhances CKD prediction across ancestries.^3^ However, the study did not investigate further the performance of their score among *APOL1* HR.

In this article, we evaluated the performance of a PRS for CKD stage 3 or higher in individuals with *APOL1* HR. We tested the PRSs using African American (AA) individuals from Bio*Me*, an electronic health record–linked diverse clinical cohort, and then validated the result in the *All of Us* Research Program data.

We first generated two new PRSs (PRS_1_ and PRS_2_) using the same method by which the most recent PRS for CKD (PRS_3_) was developed (Khan *et al.*, 2022).^3^ The first score, PRS_1_, differs from PRS_3_ in using individuals of African ancestry in Bio*Me* as the optimization cohort instead of European ancestry (Figure 1). Our second score, PRS_2_, differs from PRS_1_ in using effect size from different summary statistics. Namely, we computed PRS_2_ on the basis of summary statistics from a genome-wide association study (GWAS) for the eGFR in an African American cohort of 16,474 participants.^5^ We then tested for the association between the three scores with CKD stage 3 and higher, defined by a validated electronic algorithm in Bio*Me*.^6^ In *All of Us*, we used preidentified cases from the database on the basis of diagnostic codes. We calculated variance explained for the PRS component, using the Nagelkerke's pseudo R^2^ and adjusted odds ratios (aOR) from logistic regression models adjusted for age, sex, type 2 diabetes, and four principal ancestry components.

**Figure 1:**
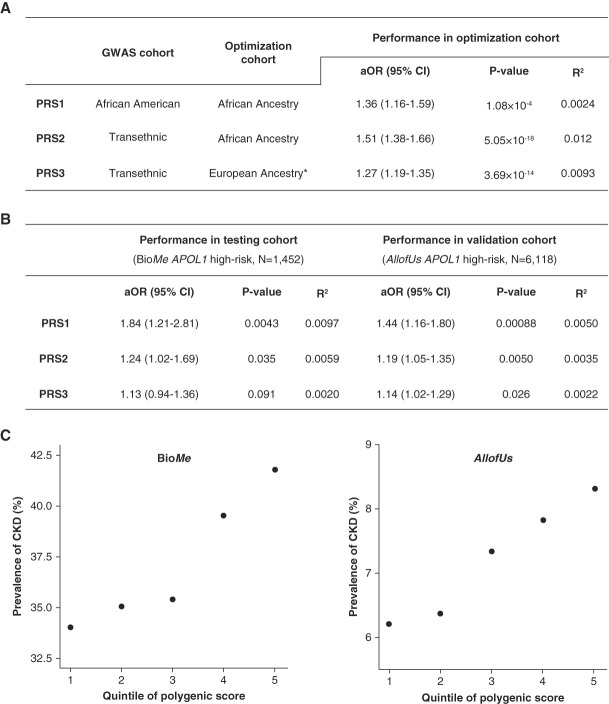
**The performance of the PRSs for CKD stage 3 and above in *APOL1* HR.** (A) The performance of the PRSs for CKD stage 3 and above in optimization cohort (AAs in Bio*Me*). (B) The performance of the PRSs for CKD stage 3 and above in testing cohort (AAs with *APOL1* HR in Bio*Me*) and in validation cohort (AAs with *APOL1* HR in *All of Us*). (C) The proportion of CKD stage 3 and above by PRS_1_ quintiles in testing (left) and validation (right) cohort. We calculated variance explained for the PRS component using the Nagelkerke's pseudo R^2^. We calculated aOR from logistic regression models adjusted for age, sex, type 2 diabetes, and four principal ancestry components. All PRSs were normalized before association testing. *PRS_3_ is a linear combination of a PRS optimized for the UK Biobank individuals of European ancestry and the *APOL1* HR genotype. AA, African American; aOR, adjusted odds ratio; *APOL1* HR, apolipoprotein L1 high-risk; CI, confidence interval; GWAS, genome-wide association study; PRS, polygenic risk score.

In the optimization cohort of 10,497 Bio*Me* AAs, PRS_1_ obtained with tuning parameters *r*^2^=0.2 and *P* = 0.3 (see the pruning and *P* value thresholding method in the study of Khan *et al.* 2022^3^) and PRS_2_ obtained with *r*^2^=0.2 and *P* = 0.1 were best associated with CKD. PRS_2_ performed better than PRS_1_ (aOR=1.51, *P* = 5.05×10^−18^, and R^2^=1.2% versus aOR=1.36, *P* = 1.08×10^−4^, and R^2^=0.93%). However, in the testing cohort of 1452 *APOL1* HR, PRS_1_ performed better than PRS_2_ (R^2^, 0.97% versus 0.59%). When we compared the performance of PRS_1_ and PRS_2_ with the most recently developed PRS (PRS_3_) for CKD across ancestry, we observed better performance of both PRS_1_ and PRS_2_ in the testing cohort (Figure 1, A and B).

We replicated the analysis in an independent validation cohort of 6118 AAs with *APOL1* HR from *All of Us* and observed a significant association of PRS_1_ and PRS_2_ with CKD (Bonferroni-corrected *P* value = 0.017). Consistently, the most significant association was observed for PRS_1_ (aOR=1.44; 95% confidence interval [CI], 1.16 to 1.80; *P* = 8.80×10^−4^). The association was weaker with PRS_2_ (aOR=1.19; 95% CI, 1.05 to 1.35; *P* = 0.005) and PRS_3_ (aOR=1.14; 95% CI, 1.02 to 1.29; *P* = 0.03) (Figure 1B).

We further examined the changes in CKD prevalence along the PRS_1_ distributions. We found that the prevalence of CKD increases from 34% in the first quintile to 42% in the last quintile in Bio*Me* and from 6% to 8% in *All of Us* (Figure 1C).

We showed that polygenic risk weakly associates with CKD in individuals with *APOL1* HR. Although no significant interactions between *APOL1* genotypes and PRS were detected (data not shown), the associations of PRS differed in *APOL1* HR versus in AAs in general. We also showed that in individuals with *APOL1* HR, polygenic scores computed from summary statistics for eGFR in AAs outperformed the most recently developed PRS_3_ (R^2^, 0.5% versus 0.2%), suggesting the polygenicity of kidney disease among *APOL1* HR is better captured by these summary statistics.

Understanding polygenic and *APOL1* HR interplay has clinical implications. *APOL1* genetic testing is now frequent, especially in transplant evaluation and direct-to-consumer testing. Thus, it is important to understand which individuals will develop the disease and polygenicity is a component of disease risk. Although the elevation of CKD risk by PRS is mild (the top quintile has 1.3 times higher risk of developing CKD than the bottom quintile), there may be value to including polygenic risk as a genetic marker, along with other nongenetic risk factors, for more optimal kidney allocation and improved pretransplant living donor counseling. However, further validation and implementation testing is needed before practical application.

## Data Availability

Previously published data were used for this study. Wuttke M, *et al.* A catalog of genetic loci associated with kidney function from analyses of a million individuals. *Nat Genet.* 51, 957–972 (2019). Pattaro C, Teumer A, Gorski M, *et al.* Genetic associations at 53 loci highlight cell types and biological pathways relevant for kidney function. *Nat Commun.* 7, 10023 (2016).
